# Co-action equilibrium fails to predict choices in mixed-strategy settings

**DOI:** 10.1038/s41598-017-19085-0

**Published:** 2018-01-15

**Authors:** Ulrich Berger

**Affiliations:** Department of Economics, WU Vienna University of Economics and Business, Welthandelsplatz 1, 1020 Vienna, Austria

## Abstract

Social projection is the tendency to project one’s own characteristics onto others. This phenomenon can potentially explain cooperation in prisoner’s dilemma experiments and other social dilemmas. The social projection hypothesis has recently been formalized for symmetric games as *co-action equilibrium* and for general games as *consistent evidential equilibrium*. These concepts have been proposed to predict choice behavior in experimental one-shot games. We test the predictions of the co-action equilibrium concept in a simple binary minimizer game experiment. We find no evidence of social projection.

## Introduction

In social psychology, social projection is the well-established tendency of people to project their own preferences, beliefs and behaviors onto other members of their own social groups^1^. This concept grew out of early research on the false consensus effect^2^ and has been extensively studied in recent decades^3^. In decision-making contexts, social projection results from evidential reasoning^4^. According to the theory of evidential decision-making, this kind of reasoning is evoked when an agent perceives other agents about whom he has no specific knowledge as belonging to the same social group as he does (his *ingroup*) and as being in the same decision-making situation. The agent then treats his own decision as diagnostic for the other agents’ decisions; i.e., he projects his own choices onto them. When transferred to a strategic context, this means that a player in a symmetric game believes that other players will choose the same (possibly mixed) strategy as she herself does.

Evidential reasoning is relatively uncontroversial if applied *after* one’s own choice has been made. However, some researchers have proposed evidential reasoning as a rational way to choose a strategy by deliberating on the consequences of one’s own hypothetical choices in combination with the resulting choices of others. According to this view, a player in a prisoner’s dilemma situation should reason that if she cooperates, her opponent will be likely to cooperate as well, whereas if she defects, he will also be likely to defect. Comparing her own payoffs in the two resulting strategy profiles, (*C*, *C*) and (*D*, *D*), will then lead the player to choose cooperation. Early arguments along these lines have been prominently—and controversially—presented by Rapoport^5^ and Hofstadter^6^.

Since cooperation is strictly dominated in the prisoner’s dilemma, evidential reasoning violates Savage’s sure-thing principle^7^. It has therefore been dismissed by Quattrone and Tversky^8^, and game theorists typically consider it to be irrational^9,10^ or a form of magical thinking^11^. Proponents of evidential reasoning, on the other hand, stress that players do not believe that their choice has any causal influence on the other player’s decision. Instead, they claim that players simply recognize that because they share a common cause (because of their similarity), in the end, their own and their opponent’s choices will be highly correlated regardless of what they choose^12^.

In a symmetric game between similar players, social projection leads selfish players to choose strategies resulting in efficient outcomes. Thus, social projection may serve as a potential explanation for empirical findings on voting behavior and for experimental choices in prisoner’s dilemma games as well as in chicken, stag-hunt and pure coordination games^12–15^.

Social projection in normal-form games has recently been independently formalized by Al Nowaihi and Dhami^16^ (see also Dhami^17^) for general games and by Sasidevan and Sinha^18^ for symmetric two-player games. Sasidevan and Dhar^19^ previously suggested it in the context of the minority game. In Al Nowaihi and Dhami^16^, the solution concept is called *consistent evidential equilibrium*, whereas Sasidevan and Dhar^19^ and Sasidevan and Sinha^18^ call it *co-action equilibrium* (CAE) and link it to the Pavlov strategy for the iterated prisoner’s dilemma^20^. CAE is a special case of consistent evidential equilibrium in which the game is symmetric with two players, both of whom use an *identity social projection function* (i.e., each believes that his opponent will play the same strategy as he himself does). The calculation of a CAE σ* in a symmetric game is simple: Each player *i* chooses a strategy *σ*_*i*_ from the common space Σ of mixed strategies such that his payoff *u*_*i*_ is maximized under the assumption that all other players choose the same strategy as he does; consequently,

$${\sigma }_{i}^{\ast }=\mathop{{\rm{\arg }}\,{\rm{\max }}}\limits_{{\sigma }_{i}\in {\rm{\Sigma }}}\,{u}_{i}({\sigma }_{i},{\sigma }_{i},\ldots ,{\sigma }_{i}\mathrm{)}.$$

As an illustration, consider the CAE of a prisoner’s dilemma scenario. We use the usual notation here: players can choose *C* (cooperate) or *D* (defect), and the payoffs are *R* (reward) for mutual cooperation, *P* (punishment) for mutual defection, *T* (temptation) for unilateral defection, and *S* (sucker’s payoff) for unilateral cooperation, where *T* > *R* > *P* > *S*. Under the social projection assumption, player 1 recognizes that a choice of *C* would result in the profile (*C*, *C*), giving him a payoff *R*, whereas choosing *D* would result in (*D*, *D*), with a payoff *P*. Since *R* > *P*, player 1 prefers to cooperate rather than defect. However, if *T* + *S* > 2*R*, then a mixed strategy can produce an even better results: If player 1 cooperates with probability *p*, then under the assumption that player 2 chooses the same mixture, his expected payoff is ${u}_{1}={p}^{2}(R+P-T-S)+p(T+S-2P)+P$. For *T* + *S* > 2*R*, this quantity is maximized at ${p}^{\ast }=\frac{T+S-2P}{\mathrm{2(}T+S-R-P)} < 1$. Thus, the CAE of a prisoner’s dilemma is (*C*, *C*) only if 2*R* ≥ *T* + *S*. If this inequality is reversed, then the efficient strategy profile, and therefore the CAE, is strictly mixed.

Sasidevan and Sinha^18^ emphasize that CAE is the first solution concept that allows for probabilistic cooperation in the prisoner’s dilemma (with 2*R* < *T* + *S*) and that this might explain why experimental cooperation rates in the prisoner’s dilemma typically decrease with increasing temptation *T*. Moreover, the CAE concept was previously introduced by Sasidevan and Dhar^19^ as a concept allowing for a mixed optimal strategy choice. Hence, the consideration of mixed strategies clearly plays an important role in the CAE concept. On the other hand, under social projection, payoff maximization is obviously much more cognitively demanding in the case of mixed strategies than for pure strategies. However, the question of whether the CAE concept is useful for explaining or predicting strategy choices in normal-form games where optimization requires mixing is ultimately an empirical one.

## Methods

### Testing the CAE prediction with the minimizer game

Although the explanatory power of the social projection hypothesis for choice behavior has been demonstrated in several social dilemma games, the ultimate test of a scientific hypothesis is a test of its ability to generate ex ante predictions for as-yet-unobserved phenomena. Both Al Nowaihi and Dhami^16^ and Sasidevan and Sinha^18^ note that the CAE solution concept is most convincing for the symmetric case with “similar” or “like-minded” players. In symmetric games, this approach typically yields a unique prediction of initial choices and might therefore serve as a parameter-free behavioral alternative to Nash equilibrium. However, the predictions of the social projection hypothesis have not yet been explored beyond the four symmetric 2 × 2 games mentioned above, and to my knowledge, they have not yet been formally tested.

Here, we present the first formal experimental test of the predictions of the CAE concept outside of the class of social dilemma games by using the *minimizer game* (MG), which requires randomization under social projection and was recently introduced in^21^ as a testbed for comparing choice predictions in one-shot games. The MG is specifically designed to circumvent the problems associated with the fact that monetary incentives are biased by social preferences. We consider the binary version of the MG here.

### Rules of the binary minimizer game

In this game, two monetary prizes, a low one *L* and a high one *H*, are offered to a group of *n* ≫ 1 players. After each player has made her choice, the *minority’s* prize is determined and is paid to one player randomly chosen from among the members of the minority. In the case of a tie, the minority is chosen by a coin flip.

Maximizing players should therefore choose the prize they think will be chosen less often by others. In the unique symmetric mixed Nash equilibrium, each player chooses the low prize *L* with probability *p*_*NE*_, which makes all players indifferent between choosing *L* and *H*. The low prize therefore must have a higher probability of being the minority’s prize. Consequently, we always have *p*_*NE*_ < 1/2.

Evidential reasoning, however, leads players to expect others to choose the same strategy as they themselves do. A player choosing a pure strategy *L* or *H* would therefore expect to end up in the majority and earn a zero payoff. The optimal choice therefore must be a mixed one. When choosing their mixtures, players will place more weight on the low prize than on the high one, thereby increasing the chance that the high prize will be the minority’s prize. For the CAE solution ${\sigma }_{i}^{\ast }=({p}_{CAE},1-{p}_{CAE})$, this means that ${p}_{CAE} > \mathrm{1/2}$.

Payoff functions and CAE solutions cannot be written down in closed form but can be readily calculated numerically: From the perspective of player *i*, for a given odd *n* and *p* ∈ [0, 1], the probability *q*(*k*) that the low prize is chosen $0\le k\le n-1$ times among the other players is given by the pmf of the corresponding binomial distribution. If the low prize is chosen $k < (n-\mathrm{1)/2}$ times among the other players, then with probability *p*, player *i* also chooses the low prize, ends up in the minority and receives an expected payoff of $L/(k+\mathrm{1)}$. If the low prize is chosen $k > (n-\mathrm{1)/2}$ times among the other players, then with probability 1 − *p*, player *i* chooses the high prize, ends up in the minority and receives an expected payoff of $H/(n-k)$. In all other cases, player *i* ends up in the majority and receives a payoff of zero. Summing the components $q(k)pL/(k+\mathrm{1)}$ and $q(k\mathrm{)(1}-p)H/(n-k)$ across their respective regions $0\le k < (n-\mathrm{1)/2}$ and $(n-\mathrm{1)/2} < k\le n-1$ yields the expected payoff *u*_*i*_(*p*, …, *p*) when player *i* chooses *p* under social projection. The CAE solution *p*_*CAE*_ is then the probability that maximizes this payoff. Similarly, the symmetric Nash equilibrium can be found by numerically equating the expected payoffs of player *i* when choosing *L* and *H*, respectively, when all other players’ choices are randomized with probability *p*.

Note that by definition, the CAE also maximizes the expected sum of the players’ payoffs. In the binary MG, this sum is either 0, *L*, or *H*. Therefore, if *H* and *L* are identical, CAE requires the probability that the payoff sum is 0, i.e., that the minority is empty, to be minimized. Thus, players should choose each option with probability *p* = 1/2. However, if *H* is much larger than *L*, CAE dictates that the probability that *H* is paid out should become large, meaning that players should choose *L* with a high probability *p*, making *H* the minority choice but simultaneously ensuring that this minority does not become empty.

**Figure 1:**
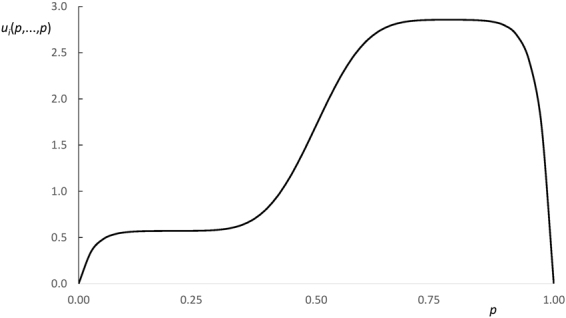
Evidential reasoner’s objective function *u*_*i*_(*p*, …, *p*) for *L* = 20, *H* = 100 and *n* = 35.

A typical objective function *u*_*i*_(*p*, …, *p*) for an evidential reasoner is depicted in Fig. 1.

When testing whether CAE successfully predicts experimental choices, the null hypothesis can be stated in both a strong and a weak form:

**(H0a, strong)** The choice frequencies are given by the CAE.

**(H0a, strong)** The choice frequencies deviate from the symmetric Nash equilibrium in the direction predicted by the CAE.

H0a is the prediction based on the assumption that all subjects use evidential reasoning. If only some subjects are assumed to do so, then H0b should still hold.

### Experiments

I conducted the minimizer game experiment at WU Vienna in 2016. A total of 434 subjects from eleven classes were invited to take part in a short in-class choice experiment. All invited subjects participated. The groups were chosen from five different undergraduate and graduate programs at WU such that none of the participants had previously taken a course on game theory. The experiment was conducted following the WULABS–Competence Center for Experimental Research ethical rules for economics experiments.

I used two treatments. Treatment 50/60 had a high ratio *L*/*H* between the two prizes (*L* = €50, *H* = €60), and treatment 20/100 had a low ratio (*L* = €20, *H* = €100); however, the mean of the two prizes was similar in both treatments. As argued above, for groups of comparable size, CAE predicts that the low prize will be chosen more often in the 20/100 treatment than in the 50/60 treatment. This prediction allows us to state a third null hypothesis:

(**H0c**) The low prize is chosen more frequently in the 20/100 treatment than in the 50/60 treatment.

**Figure 2:**
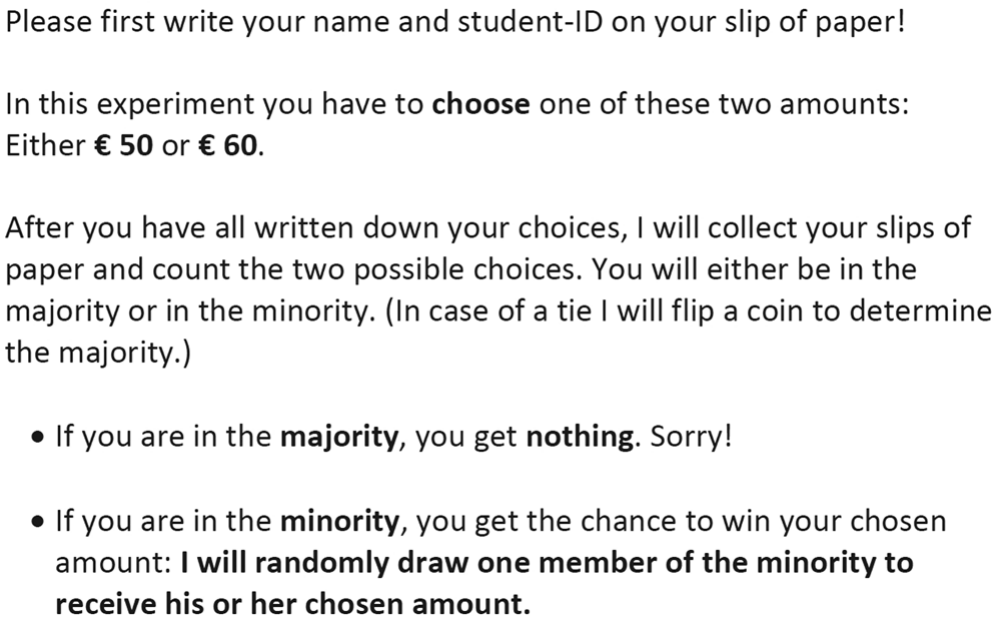
Instructions for the 50/60 treatment.

Figure 2 shows the instructions for the 50/60 treatment presented to the subjects. To ensure common knowledge of the rules of the game, the instructions were projected on the whiteboard in the classroom and read aloud.

After reading the instructions, the members of each group were given two minutes to contemplate and write down their choices. Immediately after that, the minority was determined, and the winner was drawn randomly from the minority and paid his or her chosen prize. The experiments lasted approximately five minutes, with the average payments corresponding to an hourly wage of approximately €10.

### Data availability

All data generated or analyzed during this study are included in this published article.

## Results

All results are presented in Table 1. In ten of the eleven groups, the high prize was chosen by the majority, and in the remaining group, there was a draw. In the 20/100 treatment, the tendency to choose the high prize was much more pronounced than in the 50/60 treatment. In each of the eleven groups, the choices significantly differed from the CAE prediction (according to a two-sided binomial test with a significance level of 5%) and did so by a wide margin. When groups under the same treatment were pooled using Stouffer’s weighted *Z*-test, the choices also significantly differed from the symmetric Nash equilibrium. However, even the direction of this deviation was not the one predicted by the CAE. Whereas the CAE predicted that approximately three quarters of each group would choose the low prize and the Nash prediction was slightly below one half, in ten of the eleven groups even fewer subjects chose the low prize.

**Table 1:** Summary of results.

Group	*L*/*H* prize	*n*	Low	High	*p*_*NE*_	*p*_*CAE*_	**Data**	*p*-value (NE)	*p*-value (CAE)
1	50/60	23	10	13	0.49	0.74	0.43	0.60	0.00
2	50/60	65	27	38	0.49	0.78	0.42	0.21	0.00
3	50/60	24	12	12	0.49	0.75	0.50	1.0	0.01
4	50/60	22	9	13	0.49	0.74	0.41	0.46	0.00
5	50/60	72	26	46	0.49	0.79	0.36	0.02	0.00
1–5 pooled		206	84	122	0.49	0.77	0.41	0.02	0.00
6	20/100	38	10	28	0.43	0.79	0.26	0.03	0.00
7	20/100	25	3	22	0.42	0.78	0.12	0.00	0.00
8	20/100	45	10	35	0.44	0.78	0.22	0.00	0.00
9	20/100	32	10	22	0.43	0.78	0.31	0.19	0.00
10	20/100	43	9	34	0.44	0.78	0.21	0.00	0.00
11	20/100	45	14	31	0.44	0.78	0.31	0.08	0.00
6–11 pooled		228	56	172	0.43	0.78	0.25	0.00	0.00

Consequently, both the strong and weak forms of our null hypothesis as well as the variant based on comparative statics must be rejected. The choices made in the binary minimizer game are not compatible with the social projection hypothesis as formalized by co-action equilibrium or consistent evidential equilibrium.

## Discussion

Social projection is a heuristic, and it is therefore most plausible if it does not involve cognitively demanding calculations. Although “pure-strategy social projection”, in which a player compares only *pure* symmetric strategy profiles, might explain coordination on the payoff-dominant equilibrium in pure coordination games and cooperation in prisoner’s dilemma or public goods games, it is much less plausible when extended to mixed strategies, as is inherent in co-action equilibrium. In the minimizer game, even in its simplest binary variant, pure-strategy social projection does not discriminate between the two prizes offered, but when the choice set is extended to include mixed strategies, calculation of expected payoffs under evidential reasoning seems too complicated to provide a guide for one’s choice.

There are at least three other concepts that lead to the same prediction as CAE in symmetric normal-form games and therefore in the minimizer game: *Kantian equilibrium*^22^, *homo moralis*^23^ and *team reasoning*^24^. I simply mention these concepts for completeness here; I do not intend to argue that my experimental results refute them. These concepts have not been explicitly proposed to explain or predict choices in games such as the minimizer game, which is indeed rather implausible to invoke moral reasoning or a team frame among the experimental subjects.

I wish to remark that we first used the minimizer game to test a parameter-free version of Poisson cognitive hierarchy theory and observed results that were very close to the predictions of that model^21^. For the current experiment, however, a short calculation reveals that the Poisson cognitive hierarchy model predicts *p*_*L*_ = 0.502 for both treatments, which is also far from the actual frequency of choices and deviates from the Nash prediction in the wrong direction. The same can be shown to apply to quantal response equilibrium^25^ and generalized impulse balance equilibrium^26^, which are both very close to the symmetric Nash equilibrium in our experimental game. The search for a behavioral model that is able to predict strategy choices in the minimizer game is still ongoing.
